# Current Use, Capacity, and Perceived Barriers to the Use of Extracorporeal Cardiopulmonary Resuscitation for Out-of-Hospital Cardiac Arrest in Canada

**DOI:** 10.1016/j.cjco.2020.11.005

**Published:** 2020-11-13

**Authors:** Brian Grunau, Sam D. Shemie, Lindsay C. Wilson, Katie N. Dainty, Dave Nagpal, Laura Hornby, Yoan Lamarche, Sean van Diepen, Hussein D. Kanji, James Gould, Richard Saczkowski, Steven C. Brooks

**Affiliations:** a Department of Emergency Medicine and Centre for Health Evaluation and Outcome Sciences, University of British Columbia and St Paul’s Hospital, Vancouver, British Columbia, Canada; b Division of Critical Care Medicine, McGill University, Montreal, Quebec, Canada; c Deceased Donation, Canadian Blood Services, Ottawa, Ontario, Canada; d Patient-Centred Outcomes, North York General Hospital, Toronto, Ontario, Canada; e Institute of Health Policy Management and Evaluation, University of Toronto, Toronto, Ontario, Canada; f Divisions of Cardiac Surgery and Critical Care Medicine, Western University, London, Ontario, Canada; g Children's Hospital of Eastern Ontario Research Institute, Ottawa, Ontario, Canada; h Divisions of Cardiac Surgery and Critical Care Medicine, University of Montreal, Montreal, Quebec, Canada; i Department of Critical Care and Division of Cardiology, Department of Medicine, University of Alberta, Edmonton, Alberta, Canada; j Division of Critical Care Medicine, University of British Columbia, Vancouver, British Columbia, Canada; k Department of Emergency Medicine, Dalhousie University, Saint John, New Brunswick, Canada; l Division of Cardiac Surgery, Kelowna General Hospital, Kelowna, British Columbia, Canada; m Department of Emergency Medicine, Queen’s University, Kingston, Ontario, Canada

## Abstract

**Background:**

Extracorporeal cardiopulmonary resuscitation (ECPR) is a therapeutic option for refractory cardiac arrest. We sought to perform an environmental scan to describe ECPR utilization in Canada and perceived barriers for application to out-of-hospital cardiac arrest (OHCA).

**Methods:**

This was a national cross-sectional study. We identified all cardiovascular surgery- and extracorporeal membrane oxygenation (ECMO)-capable hospitals in Canada and emergency medical services (EMS) agencies delivering patients to those centres. We requested the medical lead from each hospital’s ECMO service and each EMS agency to submit data regarding ECMO and ECPR utilization, as well as perceived barriers to ECPR provision for OHCA.

**Results:**

We identified and received survey data from 39 of 39 Canadian hospital institutions and 21 of 22 EMS agencies. Of hospitals, 38 (97%) perform ECMO and 27 (69%) perform ECPR (74% of which perform ≤5 cases per year). Of the 18 (46%) sites offering ECPR for OHCA, 8 apply a formal protocol for eligibility and initiation procedures. EMS agencies demonstrate heterogeneity with intra-arrest transport practices. The primary rationale for nontransport of refractory OHCA is that hospital-based care offers no additional therapies. Perceived barriers to the use of ECPR for OHCA were primarily related to limited evidence supporting its use, rather than resources required.

**Conclusion:**

Many Canadian cardiovascular surgery- or ECMO-equipped hospitals use ECPR; roughly half employ ECPR for OHCAs. Low case volumes and few formal protocols indicate that this is not a standardized therapy option in most centres. Increased application may be dependent on a stronger evidence base including data from randomized clinical trials currently underway.

## Résumé

**Contexte:**

La réanimation cardiorespiratoire (RCR) extracorporelle est une option thérapeutique en cas d'arrêt cardiaque réfractaire. Nous avons voulu faire une analyse contextuelle de l'utilisation de la RCR extracorporelle au Canada et des obstacles perçus quant à son emploi dans les cas d'arrêt cardiaque en dehors de l'hôpital.

**Méthodologie:**

Il s’agissait d’une étude nationale transversale. Nous avons repéré tous les hôpitaux en mesure d'effectuer des chirurgies cardiovasculaires et d'offrir l'oxygénation extracorporelle au Canada et les fournisseurs de services médicaux d'urgence (SMU) transportant les patients vers ces centres. Nous avons demandé au chef médical du service d'oxygénation extracorporelle de chacun des hôpitaux et de chacun des fournisseurs de SMU de présenter leurs données concernant l'utilisation de l'oxygénation extracorporelle et de la RCR extracorporelle, ainsi que les obstacles perçus quant à l'emploi de la RCR extracorporelle dans les cas d'arrêt cardiaque en dehors de l'hôpital.

**Résultats:**

Nous avons obtenu les données de 39 des 39 établissements hospitaliers canadiens ciblés, et de 21 des 22 fournisseurs de SMU. Parmi les hôpitaux, 38 (97%) utilisent l'oxygénation extracorporelle et 27 (69 %), la RCR extracorporelle (74 % ayant eu recours à celle-ci dans ≤ 5 cas par année). Parmi les 18 (46 %) centres offrant la RCR extracorporelle en cas d'arrêt cardiaque en dehors de l'hôpital, 8 appliquaient un protocole officiel pour l'admissibilité et les procédures de mise en place. Les fournisseurs de SMU ont montré des pratiques hétérogènes concernant la réanimation en déplacement. La principale raison justifiant de ne pas transporter les patients présentant un arrêt cardiaque réfractaire en dehors de l'hôpital est que les soins en milieu hospitalier n'offrent pas de traitements additionnels. Les obstacles perçus quant à l'emploi de la RCR extracorporelle dans les cas d'arrêt cardiaque en dehors de l'hôpital étaient principalement liés au peu de données appuyant son utilisation, plutôt qu'aux ressources nécessaires.

**Conclusion:**

De nombreux hôpitaux canadiens équipés pour la chirurgie cardiovasculaire et l'oxygénation extracorporelle utilisent la RCR extracorporelle; environ la moitié d'entre eux utilisent la RCR extracorporelle en cas d'arrêt cardiaque en dehors de l'hôpital. Le faible nombre de cas et le peu de protocoles officiels indiquent qu'il ne s'agit pas d'une option de traitement standardisée dans la plupart des centres. Une utilisation plus fréquente pourrait nécessiter davantage de données probantes, y compris les résultats des études cliniques à répartition aléatoire qui sont en cours.

Emergency medical services (EMS) attend 134 out-of-hospital cardiac arrests (OHCAs) per 100,000 adult citizens yearly in North America,^1^ a proportion of whom are young, previously healthy persons.^2^ Unfortunately, overall survival is low, with typically less than 50% achieving return of spontaneous circulation (ROSC) and only 5%-15% surviving to hospital discharge.^1^

Extracorporeal membrane oxygenation (ECMO), a form of cardiopulmonary bypass, can be used as a rescue therapy (extracorporeal cardiopulmonary resuscitation [ECPR]) in resuscitations refractory to conventional efforts.^3^ ECPR has the potential to overcome the requirement for ROSC, providing critical perfusion of the brain and other vital organs, whereas the cause for OHCA is identified and addressed. ECPR has been used for refractory cardiac arrest in the out-of-hospital and in-hospital settings.^4^^,^^5^ However, as the time from the cardiac arrest to ECMO initiation is strongly correlated with survival (with few survivors if this interval exceeds 60 minutes), achieving positive outcomes among OHCAs remains challenging.^4^^,^^5^

Current data presented by the Extracorporeal Life Support Organization demonstrate that the use of ECMO and ECPR is increasing.^6^ Within Canada, the degree to which ECPR is being used in the treatment of cardiac arrest is unclear.^7^ The multidisciplinary Canadian ECPR Research Network for OHCA (formerly called the “Canadian ECPR Research Working Group”) was formed in 2016 and set a coordinated national research agenda to study ECPR for OHCA in Canada.^8^ Based on identified knowledge gaps,^8^ an environmental scan of the Canadian setting to describe the current use of ECPR for OHCA, capacity to employ ECPR for OHCA, and perceived barriers was identified as a priority. We believed that knowledge of current practices and beliefs would assist with setting an agenda for future research, program development, and collaboration. We sought to determine whether there was sufficient application of ECPR for OHCA to justify a prospective registry, which may improve our knowledge of optimal treatment strategies.

We sought to perform an environmental scan of Canadian health care institutions and EMS agencies to describe the current use of ECMO in Canada, the rate and application of ECPR for OHCA (including whether applied on an ad hoc basis or within a formal protocol), the current number of cases treated, and the perceived barriers to broader application.

## Methods

### Study design

We performed an environmental scan of Canadian institutions to describe the current application of ECMO and ECPR in Canada, specifically for OHCA, and the perceived barriers to broader application. Environmental scans include gathering and interpreting information from the internal and external environments to inform decision-making on policy, planning, program development; data for environmental scans can be from a variety of sources including administrative data, internal reports, key informants, surveys, and interviews.^9^ Our data collection incorporated 2 approaches; we asked institutional leads to: (1) provide objective historical data about their hospital practices and protocols; and (2) describe barriers they perceived to be present at their hospitals with regard to ECPR provision.

### Study population and data collection tool

We identified 2 distinct populations for our environmental scan: hospitals and EMS agencies.

#### Hospital survey

We created a list of all Canadian adult and paediatric hospitals with cardiovascular surgery and/or ECMO services. This list was generated using information from the Canadian Society of Clinical Perfusion, the Extracorporeal Life Support Organization, and additional provincial health services websites describing hospital services. Study investigators reviewed and supplemented the list for completeness. We sent an invitation letter to the department head of the cardiovascular surgery program of each of the 39 hospitals. We requested that the clinician with the most knowledge of the hospital’s ECMO services complete the survey.

#### EMS survey

Using the list of Canadian hospitals with cardiovascular surgery and/or ECMO services, we identified all EMS agencies that transport patients to these sites. We identified these EMS agencies through internet searches and telephone calls to each region and sent an invitation letter to the medical director of each of the 22 services.

### Data collection tool development and administration

The affiliated research ethics boards of Providence Health Care and the University of British Columbia approved the study. Using previously described methodology,^10^^,^^11^ we created unique English and French language surveys for both the Hospital and EMS surveys using RedCap (Research Electronic Data Capture, Nashville, TN) (Supplemental Appendices S1 and S2). Survey questions were generated based on questions and content identified during the meetings of the Canadian ECPR Research Working Group (meeting dates May 2016 and April 2017),^8^ which included a multidisciplinary group with representation from prehospital medicine, emergency medicine, critical care, cardiac surgery, organ donation, medical ethics, neurology, nephrology, paediatrics, and anaesthesiology. Surveys included categorical multiple-choice responses and opportunities for free text responses.

The initial hospital and EMS surveys included 49 and 34 questions, respectively. Both were pilot tested by 4 physicians with ECMO/ECPR and EMS experience and 1 academic researcher. Each survey was evaluated for clinical sensibility, comprehensiveness, clarity, face and content validity, and item redundancy. Pilot testing results were incorporated into the survey design through discussion and consensus of study investigators. After modifications, the final hospital survey included 48 questions pertaining to 5 prespecified domains (ECMO and ECPR services, hospital infrastructure, ECPR patient selection, ECPR initiation practices, and perceived barriers to the provision of ECPR) and 6 questions of demographics and attitudes, for a total of 54 questions (Supplemental Appendix S1). The final EMS survey included 29 questions pertaining to 5 prespecified domains (regional characteristics, EMS characteristics, destination hospital, ECPR protocol feasibility, and perceived barriers to an ECPR protocol) and 3 demographic questions, for a total of 32 questions (Supplemental Appendix S2).

The study population was surveyed between June and October of 2019 (inclusive). Participants were sent an invitation and letter of information by e-mail that contained a link to the online survey. No incentives were provided for completion of the survey. E-mail reminders were sent to participants who had not responded 2 and 3 weeks after the initial request for survey completion was sent. To enhance the response rate, telephone calls were made to participants who had not responded to the survey 2 weeks after the second electronic reminder. A final electronic reminder was sent 6 weeks after the initial survey request.

### Definitions

Variable definitions were determined by consensus among the study group, and were outlined on the first page of the survey (Supplemental Appendices S1 and S2). An ECMO program was defined as programs having a program director, dedicated ECMO equipment, and the capability to receive transfers for ECMO management from other centres. ECPR was defined as the initiation of venoarterial (VA)-ECMO in a patient undergoing active chest compressions during cardiac arrest.^3^ A formal ECPR protocol was defined as a system of care that may include pre-established leadership, protocols, candidacy criteria, designated equipment, and guidelines. Provided the patient meets eligibility criteria, the system is designed to have the capacity to provide the service reliably. An ad hoc system of ECPR initiation was defined as the use of ECPR outside of a formal protocol, considered on a case-by-case basis, which may not always be available depending on personnel and resource availability at the time ECPR is being considered. ECPR for OHCA was defined as ECMO initiation for: (1) patients with onset of cardiac arrest in the out-of-hospital setting; (2) ECMO initiated during active chest compressions; and (3) no in-hospital periods of sustained ROSC (ie, > 20 minutes) achieved before ECMO initiation.

### Data management and statistical analysis

The analysis was descriptive. Survey data were captured and stored in RedCap (Research Electronic Data Capture) and Microsoft Excel 2011 (Microsoft Corp, Redmond, WA). Categorical responses were presented as frequencies with percentages. For each question, the proportion of nonresponse was recorded. For survey data pertaining to perceived barriers to ECPR provision, we categorized results as “no or small barrier” or “moderate to very large barrier,” and reported the proportion of sites that described each option as “moderate to very large barrier.” For hospital survey results, we divided respondents into 2 similarly sized groups based on yearly ECPR volumes (“high” vs “low” volume) and then compared the proportion of respondents who categorized the barrier as “moderate to very large” as a difference of proportions (with 95% confidence interval [CI]).

## Results

### Survey participants

The final response rate was 39/39 (100%) for the hospital survey and 21/22 (95%) for the EMS survey, with representation from 9 of 10 provinces (Supplemental Appendix S3).

### Hospital results

The 39 hospital-based respondents reported receiving training and credentials across several clinical areas of specialty including cardiac surgery (4; 10%), critical care (7; 18%), cardiac surgery and critical care (2; 5%), critical care and cardiology (1; 3%), perfusion (23; 59%), nursing (1; 3%), and respiratory therapy (1; 3%). There were 38 (97%) and 34 (87%) who believed that “ECPR may be beneficial for a subset of patients” with in-hospital and out-of-hospital cardiac arrest, respectively.

Thirty-seven (95%) of the respondents’ hospitals perform cardiac surgery, 38 (97%) perform ECMO, 14 (36%) perform cardiac transplant, and 23 (59%) implant left ventricular assist devices. Of the 38 (97%) respondents who reported their institution used both venovenous and VA-ECMO (the remaining site reported performing neither), 32 (84%) stated that they had an ECMO program; 31 (82%) and 11 (29%) treat adult and paediatric cases, respectively, and 27 (71%) reported treating refractory cardiac arrest cases with ECPR (Table 1).

**Table 1:** Characteristics and treatment practices of 38 hospitals that reported performing ECMO treatment

Variables	Level	n (%)∗
ECMO characteristics (n = 38)		
Mean number of VA-ECMO cases/year	0-5	12 (32)
	6-10	8 (21)
	11-20	7 (18)
	21-30	5 (13)
	> 30	6 (16)
Mean number of VV-ECMO cases/year	0-5	23 (61)
	6-10	8 (21)
	11-20	3 (8)
	21-30	3 (8)
	> 30	1 (3)
Physician/surgeon with focused VA-ECMO training (mis = 2)	Yes	32 (89)
ECPR characteristics (n = 27)		
Mean number of ECPR cases/year (IHCA and OHCA)	0-2	9 (33)
	2-5	11 (41)
	6-10	4 (15)
	11-20	1 (3.7)
	> 20	2 (7.4)
Number of years offering ECPR	1-2	4 (15)
	2-5	8 (30)
	6-10	6 (22)
	> 10	9 (33)
Offer ECPR for admitted IHCA		27 (100)
Formal ECPR protocol for admitted IHCAs		13/27
Offer ECPR for emergency department IHCAs		26 (96)
Formal ECPR protocol for ED IHCAs		14/26
Offer ECPR for refractory OHCA†		19 (70)
Location of ECPR initiation for ED and OHCA ECPR cases‡	Emergency department	21 (78)
	Interventional radiology suite	5 (19)
	Catheterization laboratory	13 (48)
	Operating room	11 (41)
Physician/surgeon specifically on-call for of ECPR	Yes	13 (48)
Number of physicians/surgeons in ECPR call group	1-4	8/13
	5-9	2/13
	> 10	3/13
The on-call ECPR physician/surgeon availability	Reliably available§	11/13
	Variable	2/13
Perfusionist on-call for emergent ECMO initiation		27 (100)
ECMO circuit available for ECPR? (mis = 1)	Nearly 100% of the time	25 (93)
	> 50% of the time	1 (3.7)
	≤ 50% of the time or less	0
Process to determine ECPR eligibility	Institutional criteria	7 (26)
	General guideline and ECPR‖ MD	12 (44)
	As per designated ECPR MD	3 (11)
	ICU/cardiac surgeon on-call	5 (19)
ECPR for OHCA characteristics (n = 19)		
Formal ECPR protocol for OHCA		8 (42)
Protocol includes activation before patient arrival		3 (16)
Protocol offered 24 h per day		4 (21)
ECPR eligibility, classified by temperature	Normothermia	4 (21)
	Hypothermia	5 (26)
	Both eligible	10 (53)
Number of years offering ECPR for OHCA	1-2	8 (42)
	2-5	4 (21)
	6-10	4 (21)
	> 10	3 (16)
Number of cases per year?	1-2	10 (53)
	3-5	6 (32)
	5-10	2 (11)
	> 10	1 (5.3)

ECMO, extracorporeal membrane oxygenation; ECPR, extracorporeal cardiopulmonary resuscitation; ED, emergency department; ICU, intensive care unit; IHCA, in-hospital cardiac arrest; mis, missing response; OHCA, out-of-hospital cardiac arrest; VA, venoarterial; VV, venovenous.

∗ Denominator is the total n for the section, unless specified; missing values are subtracted from the denominator.

† ECPR for refractory OHCA is defined as a cardiac arrest that occurs in the out-of-hospital setting that is transported to hospital with ongoing CPR and ECMO is initiated with ongoing chest compressions.

‡ Respondents selected all that applied.

§ Defined as able to respond within 30 minutes.

‖ A general guideline is available; however, the designated ECMO physician or surgeon must also approve.

Table 1 describes ECMO- and ECPR-related treatment practices. On average, 12 (32%) sites reported treating both < 5 venovenous cases and < 5 VA-ECMO cases per year. Of the 27 centres that reported offering any ECPR therapies, 20 (74%) treat an average of ≤5 cases per year and 12 (45%) have been offering ECPR for ≤5 years. Of those offering ECPR, 27 (100%) and 19 (70%) sites offer to in-hospital cardiac arrests (IHCAs) and OHCAs, respectively. Among centres performing ECPR for OHCA, 8 (42%) reported having a formal protocol; 3 (16%) sites activate this protocol before hospital arrival, and 4 (21%) offer this service 24 hours a day.

Table 2 describes ECPR initiation and management practices from the 27 ECPR-performing sites. Cannulation is most often performed by cardiac surgeons, with US-guided percutaneous techniques. Respondents described ECMO management location divided between medical and surgical intensive care units, led most commonly by critical care physicians, in addition to cardiac surgeons and cardiac anaesthesiologists. Distal perfusion cannulas are placed routinely by 17 of 26 (65%) sites that provided these data.

**Table 2:** ECPR initiation and management practices from 27 applicable respondents

Variable	Level	n (%)∗
Vascular access method†	Percutaneous	16 (59)
	Cut-down	10 (37)
	Hybrid percutaneous/cut-down	6 (22)
	Provider dependent	6 (22)
Vascular access methods†	U/S guidance	21 (77)
	Fluoroscopy	8 (29)
	Transesophageal echo	13 (48)
	Landmark technique	11 (40)
Vascular access proceduralist†	Anaesthesiologists	0 (0)
	Cardiac surgeon	26 (96)
	Emergency physician	0 (0)
	General surgeon	2 (7.4)
	Interventional cardiologist	9 (33)
	Medical intensivist	8 (29)
	Thoracic surgeon	1 (3.7)
	Vascular surgeon	4 (14)
ECMO cannula insertion proceduralist†	Anaesthesiologists	1 (3.7)
	Cardiac surgeon	26 (96)
	Emergency physician	0 (0)
	General surgeons	3 (11)
	Interventional cardiologist	4 (15)
	Medical intensivist	4 (15)
	Thoracic surgeon	2 (7.4)
	Vascular surgeon	0 (0)
ECPR management ward†	Medical intensive care unit	15 (55)
	Cardiac surgical intensive care unit	16 (59)
	Cardiac/coronary intensive care unit	2 (7.4)
Day-to-day ECPR management lead†	Cardiovascular surgeon	14 (51)
	Cardiac anaesthesiologist	8 (29)
	Critical care physician	25 (93)
	Cardiologist	1 (3.7)
	Patients are transferred to another centre	1 (3.7)
	Designated ECMO MD (multiple specialities)	1 (3.7)
	Cardiac critical care specialist in conjunction with cardiac surgeon	1 (3.7)
Distal perfusion cannula placement	Routinely	17 (65)
	If signs of limb ischemia develop	9 (35)
	Missing	1

ECMO, extracorporeal membrane oxygenation; ECPR, extracorporeal cardiopulmonary resuscitation.

∗ Denominator is the number of nonmissing data points.

† Selected all that apply at their institution.

**Figure 1:**
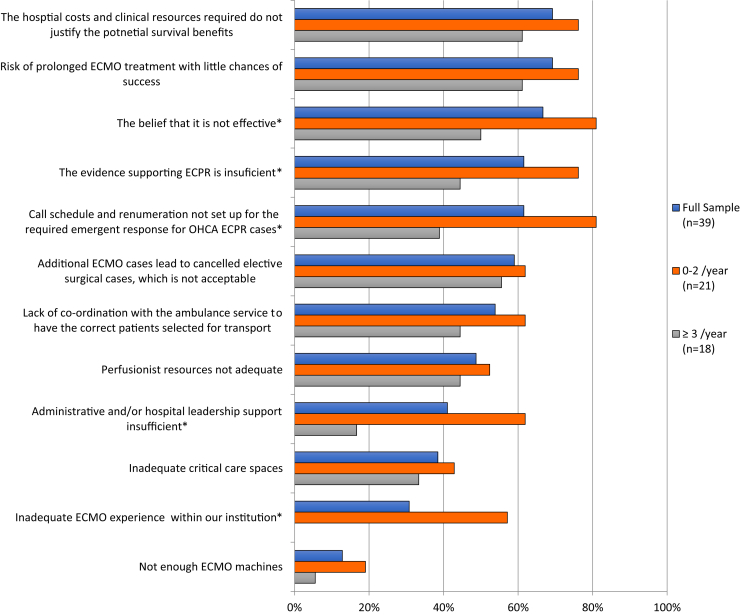
Proportion of hospital respondents who classified the barrier as “moderate” to “very large” overall and comparing sites performing 0-2 and ≥ 3 ECPR cases per year. ∗Denotes a difference between the results of sites performing 0-2 ECPR cases per year and the sites performing ≥ 3 ECPR cases per year. ECMO, extracorporeal membrane oxygenation; ECPR, extracorporeal cardiopulmonary resuscitation; OHCA, out-of-hospital cardiac arrest.

Supplemental Figure S1 displays results for survey data detailing perceived barriers to the provision of ECPR for OHCA, and Figure 1 shows the proportion of respondents who classified the individual barriers as “moderate” to “very large.” The 3 barriers identified by the highest proportion of respondents as moderate to very large pertained to ECPR effectiveness, the low chances of treatment success, and the resources not justifying the benefits; the 3 least prominent barriers pertained to critical care spaces, ECMO experience, and available ECMO machines. When comparing “high” (≥ 3 ECPR cases per year, n = 18) vs “low” (0-2 ECPR cases per year, n = 21) volume sites, low-volume sites were more likely to identify the following as a moderate to high barriers: hospital leadership support (45% difference; 95% CI: 14, 66), ECMO experience (57% difference; 95% CI: 30, 76), call schedule and remuneration (42%; 95% CI: 11, 64), supporting evidence (32% difference; 95% CI: 1, 56), and beliefs of effectiveness (31%; 95% CI: 1, 55).

### EMS results

Twenty-one EMS medical directors completed the survey, 15 (71%) of whom believed that ECPR may be beneficial for a subset of those with refractory OHCA, and 14 (67%) of whom believed that an ECPR protocol for refractory OHCA was a feasible concept. There were 5 (24%) EMS agencies that reported that ECPR for OHCA was used in some capacity within their region, 1 of which reported a formal ECPR protocol for OHCA.

The majority of EMS agencies reported that Advanced Life Support and Basic Life Support units are dispatched to OHCAs (Table 3). Respondents were asked about treatment decisions among OHCAs that proved refractory to conventional on-scene therapies. The majority of respondents indicated that these cases were transported to hospital less than 50% of cases. Of those declared dead in the prehospital setting, this decision was typically made by physician medical oversight between 16 and 30 minutes into the professional resuscitation. The most common reason for not transporting these cases to hospital was the belief that hospital-based care offered no additional benefit.

**Table 3:** Results of EMS survey

Variables	Level	n (%)∗
EMS region population	< 99,000	1 (5)
	100,000-499,999	6 (29)
	500,000-999,999	7 (33)
	> 1,000,000	7 (33)
Services dispatched to an OHCA†	Fire department first responders	20 (95)
	Bystanders‡	3 (14)
	Police	10 (48)
	BLS-trained EMS unit	20 (95)
	ALS-trained EMS unit	19 (90)
	EMS physician	0 (0)
Proportion of EMS personnel BLS-trained (%)§	0-20	1 (5)
	21-40	0 (0)
	41-60	4 (21)
	61-80	8 (42)
	81-100	6 (32)
	Missing	2
Proportion of EMS personnel ALS-trained (%)	0-20	9 (47)
	21-40	5 (26)
	41-60	4 (21)
	61-80	0 (0)
	81-100	1 (5)
	Missing	2
IAT (%)‖	< 10	4 (21)
	10-49	11 (58)
	50-90	2 (11)
	> 90	2 (11)
	Missing	2
Decision for IAT†	Paramedic discretion	12 (57)
	Physician medical oversight	15 (71)
	The universal TOR rule	12 (57)
	Initial shockable rhythms	13 (62)
	Persistent shockable rhythms	15 (71)
	Age	11 (52)
Typical time for IAT (min)¶	0-15	4 (20)
	16-30	12 (60)
	> 30	2 (10)
	Other	2 (10)
	Missing	1
Rationale for on-scene TOR†	Hospital offers no additional therapies	18 (86)
	Detrimental effect to CPR quality	10 (48)
	Risk to paramedic safety	9 (43)
	Risk to public safety	8 (38)
	Other	5 (24)
Hospital prealert for OHCA en route		21 (100)
Hospital input in regional IAT practices		9 (43)
EMS uses mechanical CPR devices		6 (29)
Hospitals within EMS region	0-10	14 (67)
	11-20	3 (14)
	> 20	4 (19)
CV surgery hospitals in EMS region	0	1 (5)
	1	13 (65)
	2	3 (15)
	5	1 (5)
	6	1 (5)
	7	1 (5)
	Missing	1
ECMO-equipped hospitals in EMS region	0	3 (14)
	1	7 (33)
	2	3 (14)
	3	2 (10)
	4	1 (5)
	Unknown	5 (24)
At least 1 hospital in region provides ECPR for OHCA		5 (24)
ECPR for OHCA provision	Ad hoc implementation	3 (75)
	A formal protocol	1 (25)
	Missing	1

ALS, Advanced Life Support; BLS, Basic Life Support; CV, cardiovascular; ECMO, extracorporeal membrane oxygenation; ECPR, extracorporeal cardiopulmonary resuscitation; EMS, emergency medical service; IAT, intra-arrest transport; OHCA, out-of-hospital cardiac arrest; TOR, termination of resuscitation.

∗ Denominator is the number of nonmissing data points.

† Respondents selected all that apply.

‡ Dispatched by a 9-1-1 Operator such as a Pulsepoint Activation.

§ Does not include firefighter agencies.

‖ Defined as: among out-of-hospital cardiac arrests that prove refractory to on-scene therapies but are then transported to hospital with ongoing CPR.

¶ Measured from the arrival of EMS personnel to scene departure.

**Figure 2:**
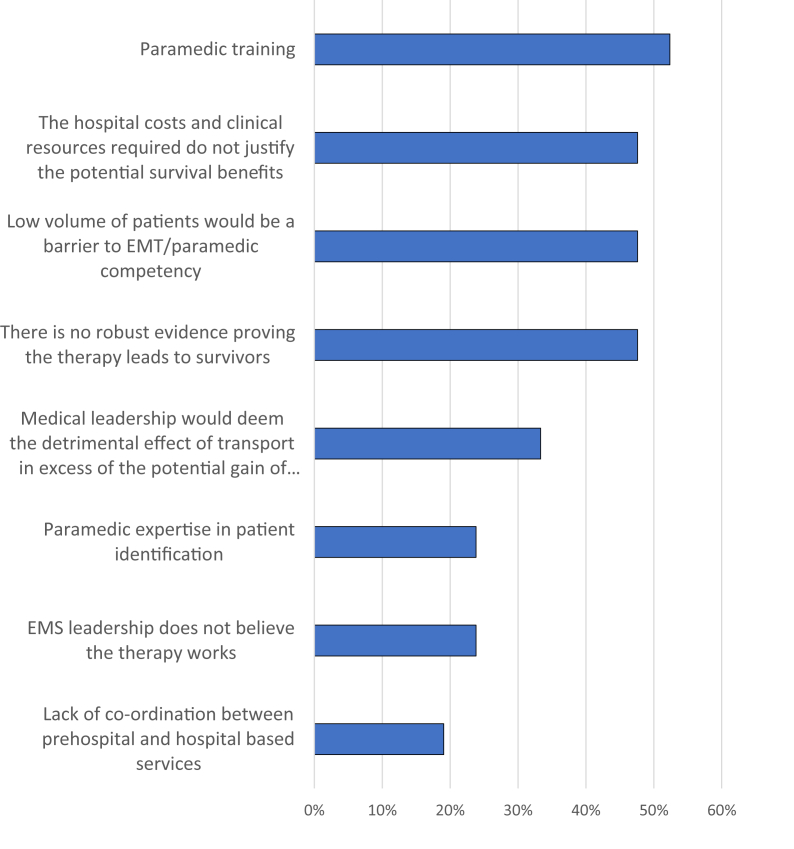
Proportion of emergency medical system (EMS) respondents who classified the barrier as “moderate” to “very large.” ECPR, extracorporeal cardiopulmonary resuscitation; EMT, emergency medical technician.

Supplemental Figure S2 displays perceived barriers to implementing an ECPR protocol within an EMS system, and Figure 2 shows the proportion of respondents who classified the individual barriers as “moderate” to “very large.” The 3 barriers identified by the highest proportion of respondents as moderate to very large pertained to paramedic training, competency in the setting of a low-volume program, and that the resources do not justify the benefits; the 3 least prominent barriers were paramedic patient identification, EMS leadership beliefs regarding effectiveness, and EMS-hospital coordination.

## Discussion

With the participation of all Canadian hospitals with cardiovascular surgery or ECMO services, as well as the emergency medical systems that serve these sites, we characterized the current ECMO utilization and ECPR practice patterns in Canada. We identified Canadian sites using ECMO therapies, with two-thirds currently offering ECPR. Although half of hospital sites offer ECPR for OHCA, volumes are relatively low and less than half of sites have formal protocols in place. Prominent hospital-based barriers to ECPR for OHCA were primarily related to the level of evidence supporting this therapy.

ECPR in Canadian institutions appears to be a relatively novel treatment modality at this time. Of the 27 sites in Canada that currently offer ECPR therapies, approximately half have used ECPR for 5 years or less. Furthermore, the total volume of ECPR experience is relatively low, with three-quarters of these sites treating 5 or less cases per year. Of ECPR-performing sites, less than half have a formal protocol in place, suggesting heterogeneity in access to this therapy, likely depending on geography, time of the day, and competing tasks. Previous data have shown that overall institutional ECMO volumes are associated with outcomes,^12^ which may also specifically apply to the management of ECPR patients, for whom ECMO initiation and ongoing management may be especially challenging and complication-prone.^13^ Low experience may in turn result in poor outcomes, which may limit enthusiasm for an ongoing ECPR program or widening indications. Clearly defined protocols, regional ECMO centres (with hub-and-spoke models or EMS diversion protocols), training, and simulation may mitigate the disadvantages inherent with low patient volumes.^7^

In comparison with IHCAs, appropriately selected patients with OHCA may be better ECPR candidates because they are generally younger, healthier, have better cardiac arrest prognostic features,^14^ and more often present with sudden unexpected manifestations of acute coronary syndromes, all of which have been predictive of improved outcomes.^4^^,^^15^ The out-of-hospital setting also includes a higher absolute number of cardiac arrests.^2^ However, ECPR outcomes are highly correlated with the cardiac arrest-to-ECMO interval, with few survivors if this interval exceeds 60 minutes.^5^ For OHCAs, achieving ECMO initiation within this window is challenging due to hospital transport time requirements, even within a formal protocol involving prehospital and hospital coordination for patient identification and team activation.^7^ Our results show that ECPR is being used for OHCA in 19 hospital sites in Canada, albeit in low volumes, with the majority treating 5 or less cases per year. Similar to sites employing ECPR for IHCA, less than half use a formal protocol, and few activate the team before patient hospital arrival. Although 8 hospital sites described a formal ECPR protocol for OHCA, this was only reported by 1 EMS service, indicating that EMS agencies in the remaining 7 sites may not be program partners. Whereas the provision of ECPR on a case-by-case basis as resources are available may be appropriate for infrequent utilization, consistent provision of ECPR services for a defined patient group may require the development of regional protocols, including prehospital- and hospital-based collaboration, to achieve ECPR access for appropriate candidates with desired arrest-to-ECMO metrics.^7^

ECPR cannulation in Canada is led primarily by cardiac surgery; however, ongoing management is divided between medical critical care wards and cardiac surgery intensive care units. Day-to-day patient management appears to be led by cardiac surgeons in half of centres, but in nearly all centres, critical care physicians play a lead or co-leadership role in the management of these patients. This may reflect the increasing importance of the ECMO team management of these complex patients, with critical care playing an increasingly important role.

Our data on perceived hospital barriers to the provision of ECPR for OHCA demonstrated several themes. First, the most prominent barriers identified were related to the evidence supporting the use of ECPR for OHCA, whereas hospital support, perfusionist resources, and infrastructure (including equipment, call schedules, and critical care spaces) were less commonly identified as an important barrier, which was surprising given the resource-intensive nature of this therapy. Resources may not be strained with currently low ECMO volumes; however, if new evidence supports the expansion of ECMO indications, these attitudes could change. When we compared the proportion of respondents classifying barriers as moderate to very large, between “low” vs “high” volume centres, the largest divergence was the barrier of institutional ECMO experience, which would be an important consideration for any plans to expand the application of this complex and technically difficult therapy. There was also a significant difference in the belief of effectiveness, which likely plays a large role in the volume of ECPR utilization at these institutions.

Canadian EMS agencies within regions offering cardiovascular surgical services appear willing and relatively well equipped to partner with hospital-based ECPR programs. Multiple units are dispatched to each OHCA, and nearly all include Advanced Life Support personnel. In many systems, a substantial proportion of cases are currently transported with ongoing resuscitation to hospital, which makes candidacy assessment more straightforward. Among regions that are terminating resuscitation in the prehospital setting, the most prominent rationale is that hospital-based care offers no additional benefit—a rationale that may shift if an ECPR protocol was introduced. Prominent barriers were related to resource implications (training and protocol resources), which may reflect the daunting task of training all paramedics in a region in a new protocol.

Our results are consistent with a study from the United States, which surveyed hospitals that submitted ECPR cases to the extracorporeal life support organization registry, to determine the utilization of ECPR in emergency departments.^16^ They reported that 36 US centres used ECMO in the emergency department, with 65% of programs less than 5 years of age, and 60% of programs performing ≤3 cases per year. A minority of programs had formal inclusion criteria.

ECPR is a resource-intensive intervention,^17^ and establishing a formal protocol at an institution, including the requisite protocol development and training, will have upfront and ongoing costs. Although ECPR for OHCA has been shown to be lifesaving and cost-effective with the right patient selection,^17^ there may be added complexities and nonfinancial costs associated with achieving successful program outcomes. One high-volume program in Minnesota has yielded impressive results with 42% of cases meeting their criteria (initial ventricular fibrillation rhythm with age 18-75) leaving hospital with favourable neurologic outcomes.^18^ Their results have shown that these patients require a very high level of care and often demonstrated prolonged periods until awakening.^13^ A small number of critical care cardiologists managed all cases, from performing the initial cannulation to hospital discharge. Other centres have had difficulties replicating these results,^19^, ^20^, ^21^ which may be due to the specialized and low-volume nature of this complex therapy. Similar to overall ECMO management, a requisite volume of cases (possibility with a small team of specialized clinicians managing all cases) may be required to yield successful results.

We sought to describe the current ECMO practices in Canada, and barriers to ECPR provision, to assist with setting an agenda for future research, program development, and collaboration, and to determine whether a prospective registry would be warranted. There appears to be an opportunity for national collaborative efforts with regard to program and protocol development, which may be especially beneficial for low-volume centres that may not have the resources or volumes to create these processes de novo. Whereas we were not previously aware that so many centres were using ECPR for OHCAs, a prospective registry may be beneficial to monitor care processes and outcomes, with the goal of quality improvement. Small prospective studies may also be feasible. On the basis of these data, the Canadian ECPR Research Network for OHCA has created an online platform for discussion and protocol sharing.

Further evidence to define the benefit of ECPR for OHCA is required,^19^^,^^22^ which was reflected in the results of our study. A clinical trial in Prague that aims to enrol 170 patients,^23^ as well as a smaller study in Vienna,^24^ will provide high-quality data comparing the strategies of (1) on-scene resuscitation vs (2) transport to hospital for ECPR initiation. These studies will be informative for systems that prioritize on-scene resuscitation. The INCEPTION^25^ (the Netherlands) and ARREST^26^ (Minnesota) clinical trials randomize patients in refractory arrest at hospital arrival, which will provide important data that can be applied to systems that tend to transport these phenotypes to hospital with ongoing resuscitation efforts.

### Limitations

This study relied on data of institutional practices and protocols provided by representative respondents. The perceived barriers to ECPR provision may not have accurately represented the average opinion of hospital staff or true resource limitations. Our survey was created for this study and has not been validated previously. There was no existing survey available on this topic, and therefore we had no choice but to develop one tailored to the objectives of the study.

## Conclusions

Many Canadian cardiovascular surgery- or ECMO-equipped hospitals use ECPR, roughly half of which employ ECPR for OHCAs. Low case volumes and few formal protocols indicate that this is not a standardized therapy option in most centres. Increased application may be dependent on a stronger evidence base including data from randomized clinical trials currently underway.
