# Supplementary material for: Current Use, Capacity, and Perceived Barriers to the Use of Extracorporeal Cardiopulmonary Resuscitation for Out-of-Hospital Cardiac Arrest in Canada
Source: CJC Open. 2020 Nov 13;3(3):327–36. doi: 10.1016/j.cjco.2020.11.005 (PMC7985000; doi:10.1016/j.cjco.2020.11.005)
Supplement: Supplemental Appendix S1 [file mmc1.pdf]

Canadian ECPR Survey

English version ***La version française suit***

ECMO-CPR for OHCA: Hospital ECMO Lead

Thank you for agreeing to participate in this survey! We hope to survey each Canadian hospital with cardiovascular surgery capacity to determine practices with regards to ECPR provision for out-of-hospital cardiac arrest. We have sent this survey to the physician, surgeon, or perfusionist in your hospital identified as the lead for ECMO services, however if you believe it is more appropriate to be filled out by another colleague please pass along this survey and email Lindsay.Wilson@blood.ca.

The survey will allow you to exit and return later if you wish.

Please use the following definitions for questions in the survey.

Definitions:

• ECMO: mechanical circulatory support to support heart and/or lung function (includes VA and VV ECMO); does not refer to routine intra-operative management of cardiac surgery

• ECPR: the initiation of VA-ECMO in a patient undergoing active chest compressions during cardiac arrest

• "ECPR for OHCA" fulfills all of the following: (i) the patient had a cardiac arrest in the out-of-hospital setting (ii) ECMO was initiated during active chest compressions (iii) the patient had no periods of sustained ROSC (ie. > 20 min) prior to ECMO initiation (Note: a patient had an OHCA and achieved ROSC which was sustained, then re-arrested during some point after hospital arrival, this is considered ECPR for in-hospital cardiac arrest)

• A formal protocol: A system of care which may include pre-established leadership, protocols, candidacy criteria, designated equipment, and guidelines. Provided the patient meets eligibility criteria, the system is designed to have the capacity to provide the service reliably.

• An ad hoc scenario: The occasional use of ECPR for OHCA within an institution without a formal protocol. Use of ECPR for OHCA may be determined by a case-by-case basis and may not always be available depending on personel and resource availability at the time ECPR is being considered.

Version française ***The English version precedes***

La RCR-ECMO pour les ACEH - sondage à l'intention des responsables de l'ECMO dans les hôpitaux

Merci d’avoir accepté de participer à ce sondage! Nous espérons sonder chaque hôpital canadien doté d’un service de chirurgie cardiovasculaire afin de déterminer les pratiques relatives à la RCR extracorporelle chez les personnes qui font un arrêt cardiaque en dehors du milieu hospitalier (ACEH). Nous avons envoyé ce questionnaire au médecin, chirurgien ou perfusionniste responsable des services d’ECMO dans votre hôpital. Toutefois, si vous croyez que l’un de vos collègues est mieux placé que vous pour y répondre, veuillez le lui transmettre et envoyer un courriel à Lindsay.Wilson@blood.ca.

Vous pouvez interrompre le sondage en tout temps et y revenir plus tard.

Veuillez vous référer aux définitions suivantes pour répondre aux questions

Définitions:

• ECMO : technique de circulation mécanique qui offre une assistance cardiaque ou respiratoire [comprend l’ECMO veino-artérielle (ECMO-VA) et l’ECMO veino-veineuse (ECMO-VV)]; ne fait pas référence à la prise en charge peropératoire habituelle de la chirurgie cardiaque

• RCR extracorporelle : amorce de l’ECMO-VA chez un patient sur lequel on pratique des compressions thoraciques pendant un arrêt cardiaque

• La définition de la « RCR extracorporelle en cas d’ACEH » englobe tout ce qui suit : (i) le patient a eu un arrêt cardiaque en dehors du milieu hospitalier; (ii) l’ECMO a été amorcée pendant les compressions thoraciques; (iii) le patient n’a eu aucune période soutenue de retour à une circulation spontanée (ROSC) (c.-à-d. > 20 min) avant l’amorce de l’ECMO. (Remarque : lorsqu’un patient fait un ACEH, qu’il y a reprise de la circulation spontanée mais qu’un autre arrêt cardiaque survient après son arrivée à l’hôpital, le traitement prodigué s’appelle « RCR extracorporelle en cas d’arrêt cardiaque à l’hôpital ».

• Protocole officiel : système de soins qui peut comprendre une direction préétablie, des protocoles, des critères de sélection, de l’équipement désigné et des lignes directrices. Dans la mesure où le patient répond aux critères d’admissibilité, le système est conçu pour pouvoir offrir le service de façon fiable.

• Situation ponctuelle : utilisation occasionnelle de la RCR extracorporelle lors d’un ACEH dans un établissement qui n’a pas de protocole officiel. Elle peut être déterminée au cas par cas et, selon les ressources et le personnel disponibles au moment où la RCR extracorporelle est envisagée, elle peut ne pas être possible.

Demographics / Données démographiques

Please enter your name ________________________________

Veuillez inscrire votre nom.

What is the name of your hospital? ________________________________

Quel est le nom de votre hôpital?

What is your position in the hospital (with respect to ECMO or cardiac care) ________________________________
(For example, "head of cardiovascular surgery", "Hospital ECMO director", Cardiovascular Surgeon, Cardiologist, Perfusionist, Critical Care Specialist, etc.   / Par exemple, chef de la chirurgie cardiovasculaire, directeur de l'ECMO de l'hôpital, chirurgien cardiovasculaire, cardiologue, perfusionniste, spécialiste en soins intensifs, etc.)

Quel poste occupez-vous à l'hôpital (dans le context de l'ECMO ou des soins cardiaques)?

What is your specialty. Please select all that apply.

Quelle est votre spécialité? Cochez toute réponse pertinente.

- ☐ Cardiac Surgeon / Chirurgien cardiaque
- ☐ Critical care physcian / Médecin de soins critiques
- ☐ Anesthesiologist / Anesthésiologiste
- ☐ Cardiologist / Cardiologue
- ☐ Other / Autre

(Select all that apply / Cochez toute réponse pertinente)

What is your specialty? ________________________________

Si vous avez répondu >, veuillez indiquer votre spécialité?

Do you believe that ECPR may be beneficial for a subset of patients with in-hospital cardiac arrest?

Croyez-vous que la RCR extracorporelle est bénéfique pour un sous-ensemble de les patients qui font un arrêt cardiaque à l'hôpital?

- ◯ Yes / Oui
- ◯ No / Non

Do you believe that ECPR may be beneficial for a subset of patients with out-of-hospital cardiac arrest?

Croyez-vous que la RCR extracorporelle est bénéfique pour un sous-ensemble de les patients qui font un arrêt cardiaque à l'extérieur de l'hôpital?

- ◯ Yes / Oui
- ◯ No / Non

ECMO & ECPR Services / Services d'ECMO et de RCR extracorporelle

Does your hospital offer any of the following services (at any volume)? Please select all that apply.

Votre hôpital offre-t-il un ou plusieurs des services suivants (peu importe le volume)? Cochez toute réponse pertinente.

☐ Cardiac surgery / Chirurgie cardiaque
☐ Cardiac transplant / Transplantation cardiaque
☐ VA ECMO / ECMO-VA
☐ VV ECMO / ECMO-VV
☐ Left Ventricular Assist Device Implantation / Installation d'un système d'assistance cardiaque
☐ ECPR / RCR extracorporelle
(Select all that apply / Cochez toute réponse pertinente)

Does your institution have an ECMO program?

Votre établissement a-t-il un programme d'ECMO?

◯ Yes / Oui
◯ No / Non
([ECMO programs typically have a program director, dedicated equipment, and receive transfers from other centers / un programme d'ECMO comporte généralement un directeur de programme et de l'équipement spécialisé et accepte des transferts d'autres centres])

On average, how many VA-ECMO patients are treated per year?

En moyenne, combien de patients par année font l'objet d'un traitement ECMO-VA?

◯ 0-5
◯ 6-10
◯ 11-20
◯ 21-30
◯ >30
(Includes all patinets treated with VA-ECMO, whether or not cardiac arrest patients / Inclure tous les patients ayant bénéficié d'un traitement ECMO VA, que ce soit en situation d'arrêt cardiaque ou non)

On average, how many VV-ECMO patients are treated per year?

En moyenne, combien de patients par année font l'objet d'un traitement ECMO-VV?

◯ 0-5
◯ 6-10
◯ 11-20
◯ 21-30
◯ >30

Which of the following do you treat with ECMO? Please seletct all that apply.

À quel groupe de patients offrez-vous l'assistance par ECMO? Cochez toute réponse pertinente.

☐ Adults (18 years of age and older) / Adultes (18 ans et plus)
☐ Pediatric / Patients pédiatriques
(Select all that apply / Cochez toute réponse pertinente)

On average how many ECPR cases (in hospital or out of hospital arrests) are treated per year?

En moyenne, combien de patients par année sont traités au moyen de la RCR extracorporelle (arrêts cardiaques en milieu hospitalier ou à l'extérieur)?

◯ 0-2
◯ 2-5
◯ 6-10
◯ 11-20
◯ >20
◯ I don't know / Je ne sais pas

Who can we contact who would know? Please write name and contact person from your institution who would know:

Veuillez indiquer le nom et les coordonnées d'une personne votre établissement qui pourrait connaître une telle Information:

____

How many years have you been offering ECPR at your hospital in any capacity?

Depuis combien d'années votre hôpital offre-t-il la RCR extracorporelle sous quelque forme que ce soit?

◯ 1-2
◯ 2-5
◯ 6-10
◯ > 10 years / > 10 ans

Does your institution ever offer ECPR for admitted patients (includes wards, ICU, cath lab) with refractory cardiac arrest that occurs within the hospital?

Vous arrive-t-il d'administrer la RCR extracorporelle aux patients admis qui font un arrêt cardiaque réfractaire au sein de votre établissement (y compris dans les salles de malades, à l'unité de soins intensifs et au laboratoire de cathétérisme)?

◯ Yes / Oui
◯ No / Non

Is ECPR for admitted patients performed within a formal protocol?

Lorsque vous pratiquez la RCR extracorporelle sur des patients admis, est-ce dans le cadre d'un protocole officiel?

◯ Yes / Oui
◯ No / Non

Does your institution ever offer ECPR for emergency department patients with refractory cardiac arrest that occurs within the hospital?

Vous arrive-t-il d'offrir la RCR extracorporelle aux patients des services d'urgence qui font un arrêt cardiaque réfractaire au sein de votre établissement?

◯ Yes / Oui
◯ No / Non

Is ECPR for emergency department arrests performed within a formal protocol?

Chez les patients en arrêt cardiaque du service des urgences, la RCR extracorporelle est-elle pratiquée selon un protocole officiel?

◯ Yes / Oui
◯ No / Non

Has your institution performed ECPR for out-of-hospital cardiac arrests who were transported to your facility with ongoing CPR?

Votre établissement a-t-il pratiqué la RCR extracorporelle sur des patients ayant fait un arrêt cardiaque extrahospitalier ayant été transportés dans votre établissement pendant que des manœuvres de réanimation étaient effectuées?

◯ Yes / Oui
◯ No / Non

For out-of-hospital cardiac arrests treated with ECPR by your institution, are these:

Dans votre hôpital, la RCR extracorporelle pratiquée pour traiter les patients ayant fait un arrêt cardiaque extrahospitalier est effectuée:

◯ Normothermic / en normothermie
◯ Hypothermic / en hypothermie
◯ Both / les deux

How many years have you been offering ECPR to out-of-hospital cardiac arrests (in any capacity)?

Depuis combien d'années offrez-vous des services de RCR extracorporelle pour traiter les cas d'arrêt cardiaque extrahospitalier (sous quelque forme que ce soit)?

- ◯ 1-2
- ◯ 2-5
- ◯ 6-10
- ◯ > 10 years / >10 ans

How many out-of-hospital cardiac arrests are treated with ECPR per year?

Combien de cas d'arrêt cardiaque extrahospitalier sont-ils traités au moyen de la RCR extracorporelle chaque année?

- ◯ 1-2
- ◯ 3-5
- ◯ 5-10
- ◯ >10

Is the provision of ECPR for out-of-hospital cardiac arrest within:

Comment se fait la prestation de la RCR extracorporelle pour les arrêts cardiaques extrahospitaliers?

- ◯ A formal protocol / Selon un protocole officiel
- ◯ Ad hoc implementation / De façon ponctuelle
- ◯ Unknown / Je ne sais pas

Please describe your system:

Veuillez décrire votre système:

____

For your ECPR formal protocol for out-of-hospital cardiac arrest. Please select all that apply.

Concernant votre protocole officiel de RCR extracorporelle pour les arrêts cardiaques extrahospitaliers (cochez toute réponse pertinente). Cochez toute réponse pertinente.

- ☐ The team mobilizes prior to patient arrival / l'équipe se mobilise avant l'arrivée du patient
- ☐ The protocol is offered 24/7 / le protocole est offert 24 h/24
- ☐ Other/ autre

(Select all that apply / Cochez toute réponse pertinente)

Please clarify "other":

Si vous avez répondu autre, veuillez préciser:

____

For ECPR initiation of ED or out-of-hospital arrests, patients are initiated on ECMO in what location? Please select all that apply.

Lorsqu'on amorce la RCR extracorporelle sur un patient qui a fait un arrêt cardiaque au service des urgences ou à l'extérieur de l'hôpital, à quel endroit démarre-t-on l'ECMO? Cochez toute réponse pertinente.

- ☐ Emergency department / Service des urgences 2, Intensive care unit / Unité de soins intensifs
- ☐ Interventional radiology suite / Salle de radiologie interventionnelle
- ☐ Catheterization laboratory / Laboratoire de cathétérisme
- ☐ Operating room / Salle d'opération
- ☐ Other / Autre

(Select all that apply / Cochez toute réponse pertinente)

Please indicate location of ECPR initiation:

Si vous avez répondu >, veuillez indiquer où la RCR extracorporelle est pratiquée dans votre établissement:

____

Hospital Infrastructure / Infrastructure de l'hôpital

Is there a physician/surgeon who is on-call specifically for emergent initiation of ECPR?

Y a-t-il un médecin ou un chirurgien qui est spécialement de garde pour les RCR extracorporelles urgentes?

- ◯ Yes / Oui
- ◯ No / Non

(Note: the physician may have other duties to perform during this period, however is specifically identified as the person to call for ECPR / Remarque: le médecin peut avoir d'autres tâches à effectuer durant cette période; il est toutefois désigné comme personne à appeler pour une RCR extracorporelle)

How many physicians/surgeons are on your ECPR call list?

Combien de médecins ou de chirurgiens sont-ils inscrits sur votre liste d'appel pour les RCR extracorporelles?

____

Is this physician/service:

Quelle est la disponibilité du médecin ou du service?

- ◯ Reliably available on an emergent basis (ie. the majority of the time can respond within 30 minutes) / Il est prêt à intervenir en cas d'urgence (c. à d. dans un délai de 30 minutes la majorité du temps)
- ◯ Availability highly dependent on their other duties / La disponibilité du médecin ou du service est fortement tributaire des autres tâches ou activités

Is there a perfusionist on-call for whom duties include emergent initiation of ECMO?

Y a-t-il un perfusionniste de garde dont les responsabilités incluent l'amorce immédiate d'une ECMO?

- ◯ Yes
- ◯ No

Are there physicians in your institution with special training and expertise in the use of VA ECMO?

Dans votre établissement, y a-t-il des médecins qui ont une formation particulière et une expertise en ECMO-VA?

- ◯ Yes / Oui
- ◯ No / Non
- ◯ Unsure / Incertain

How often is an ECMO machine available for ECPR?

Dans quelle mesure un appareil d'ECMO est-il disponible pour pratiquer une RCR extracorporelle?

- ◯ Nearly 100% of the time / Près de 100 % du temps
- ◯ >50% of the time / > 50 % du temps
- ◯ 50% or the time or less / 50 % du temps ou moins

Patient Selection / Sélection des patients

By what process does your institution evaluate eligibility of potential ECPR candidates?

Par quel mécanisme votre établissement évalue-t-il l'admissibilité des candidats à la RCR extracorporelle?

- ○ A clear institutional criteria within a formal ECPR protocol / Un protocole de RCR extracorporelle official comprenant des critères institutionnels clairs
- ○ A general guideline and call the designated on-call ECMO specialist / Une ligne directrice générale qui prévoit l'appel du spécialiste de l'ECMO qui est de garde
- ○ No guideline but a designated ECMO specialist who decides / Aucune ligne directrice, mais un spécialiste de l'ECMO désigné qui prend les décisions
- ○ No designated ECMO specialist but we call ICU/cardiac surgeon on-call / Nous n'avons aucun spécialiste de l'ECMO désigné, mais nous appelons les soins intensifs ou le chirurgien cardiaque de garde
- ○ Other / Autre

Please clarify "other":

Si vous avez répondu >, veuillez préciser: __________________________________

Please list your ECPR criteria or guideline

Veuillez indiquer les critères ou les lignes directrices de votre établissement en matière de RCR extracorporelle: __________________________________

ECPR Initiation / Démarrage de la RCR extracorporelle

How is vascular access for ECPR typically achieved? Please select all that apply:

En règle générale, comment accède-t-on aux vaisseaux pour amorcer la RCR extracorporelle? Cochez toute réponse pertinente.

- ☐ Percutaneous / Par voie percutanée
- ☐ Cut-down / Par dénudation
- ☐ Hybrid percutaneous/cut-down / Par accès hybride percutané/dénudation
- ☐ Provider dependent / La technique dépend du médecin

(Select all that apply / Cochez toute réponse pertinente)

Vascular Access is assisted by (Please select all that apply):

L'accès vasculaire est facilité par (Cochez toute réponse pertinente):

- ☐ U/S guidance / guidage échographique
- ☐ Fluoroscopy / radioscopie
- ☐ Transesophageal Echo / échocardiographie transœsophagienne
- ☐ We perform vascular access with landmark technique / nous utilisons une technique novatrice
- ☐ Other / Autre

(Select all that apply / Cochez toute réponse pertinente)

Please explain "other"

Si vous avez répondu >, veuillez expliquer __________________________________

The role of achieving arterial vascular access is the role of (please select all that apply):

À qui revient le rôle d'installer un accès vasculaire? (Cochez toute réponse pertinente)

☐ Emergency physicians / Médecins urgentistes
☐ Cardiac surgeons / Chirurgiens cardiaques
☐ Interventional cardiologists / Cardiologues d'intervention
☐ Medical intensivists / Médecins intensivistes
☐ Vascular surgeons / Chirurgiens vasculaires
☐ Other / Autre
(Select all that apply / Cochez toute réponse pertinente)

Please list who achieves arterial access

Veuillez préciser qui est chargé de l'accès artériel

____

ECMO cannulae insertion is the role of (please select all that apply):

À qui revient le rôle d'installer le cathéter pour l'ECMO (cochez toute réponse pertinente)?

☐ Emergency physicians / Médecins urgentistes
☐ Cardiac surgeons / Chirurgiens cardiaques
☐ Interventional cardiologists / Cardiologues d'intervention
☐ Medical intensivits / Médecins intensivistes
☐ Vascular surgeons / Chirurgiens vasculaires
☐ Other / Autre
(Select all that apply / Cochez toute réponse pertinente)

Please list who inserts ECMO cannulae:

Veuillez indiquer qui est responsable du cathétérisme pour l'ECMO:

____

Ongoing medical management of ECPR patients typically occurs in the (please select all that apply):

La prise en charge médicale continue des patients ayant fait l'objet d'une RCR extracorporelle a lieu généralement à (cochez toute réponse pertinente):

☐ Medical intensive care unit / l'unité médicale de soins intensifs
☐ Cardiac surgical intensive care unit (ie. the location that post-op surgical patients are managed) / l'unité de soins intensifs en chirurgie cardiaque (là où sont pris en charge les patients après la chirurgie)
☐ The cardiac intensive care unit / the coronary care unit / l'unité de soins intensifs en cardiologie / l'unité de soins coronariens
☐ Other / Autre
(Select all that apply / Cochez toute réponse pertinente)

Please list location:

Veuillez indiquer le lieu de la prise en charge:

____

Day-to-day medical management of the ECPR patient is directed by (please select all that apply):

La jour à jour prise en charge médicale des patients qui font l'objet d'une RCR extracorporelle est dirigée par (cochez toute réponse pertinente):

☐ Cardiovascular surgeon / un chirurgien cardiovasculaire
☐ Cardiac Anesthesiologist / un anesthésiologiste cardiaque
☐ General Critical Care Physician / un médecin généraliste spécialisé en soins intensifs
☐ Cardiologist / un cardiologue
☐ Other / Autre
(Select all that apply / Cochez toute réponse pertinente)

Please explain "other":

Si vous avez répondu >, veuillez indiquer de qui il s'agit:

____

When is a distal perfusion cannula placed in ECPR patients?

Dans quelles circonstances installe-t-on une canule de perfusion distale chez les patients en RCR extracorporelle?

- ◯ Routinely / Cela est fait de façon systématique
- ◯ Only if signs of limb ischemia develop / Nous le faisons seulement si nous observons des signes d'ischémie des membres

How do you determine that limb ischemia has developed?

Comment faites-vous pour déterminer qu'il y a ischémie des membres?

____

Which of the following are perceived barriers to the provision of ECPR for out-of-hospital cardiac arrest (OHCA) at your hospital? (whether or not you offer this service).

Indiquez dans quelle mesure les éléments suivants sont perçus comme des obstacles à la prestation de la RCR extracorporelle pour traiter les cas d'arrêt cardiaque extrahospitalier (ACEH) dans votre hôpital (que ce service y soit offert ou non).

	No Barrier / Aucun obstacle	Small Barrier / Mineur	Moderate Barrier / Modéré	Large Barrier / Important	Very Large Barrier / Obstacle majeur
The belief that it is not effective / On ne croit pas à l'efficacité de ce traitement	○	○	○	○	○
The evidence supporting ECPR is insufficient / Les données probantes à l'appui de ce traitement ne sont pas suffisantes	○	○	○	○	○
The hospital costs and clinical resources required do not justify the potential survival benefits / Les avantages du traitement sont insuffisants par rapport aux ressources nécessaires	○	○	○	○	○
Call schedule and remuneration not set up for the required emergent response for OHCA ECPR cases / Les tours de garde et la rémunération ne sont pas organisés pour assurer l'intervention immédiate que requiert ce service	○	○	○	○	○
Perfusionist resources are not adequate / Les ressources en perfusion sont insuffisantes	○	○	○	○	○
Not enough ECMO machines / Il n'y a pas assez d'appareils d' ECMO	○	○	○	○	○
Inadequate critical care spaces / Les espaces réservés aux soins□intensifs sont insuffisants	○	○	○	○	○

Additional ECMO cases lead to cancelled elective surgical cases, which is not acceptable / Les cas d'ECMO additionnels entraînent l'annulation de chirurgies non urgentes, ce qui est inacceptable	○	○	○	○	○
Risk of prolonged ECMO treatment with little chances of success / Le risque associé à un traitement d'ECMO prolongé par rapport aux faibles chances de réussite	○	○	○	○	○
Lack of co-ordination with the ambulance service to have the correct patients selected for transport / La possibilité que les bons patients ne soient pas sélectionnés pour le transport à cause d'un manque de coordination avec le service ambulancier	○	○	○	○	○
Inadequate ECMO experience within our institution / Le manque d'expérience en ECMO au sein de notre établissement	○	○	○	○	○
Administrative and/or hospital leadership support insufficient / Le manque de soutien administratif ou d'appui de la direction de l'hôpital	○	○	○	○	○

Please identify other key barriers

Veuillez indiquer d'autres obstacles d'importance ______________________________

Thank you for participating in this survey!

Merci d'avoir répondu à ce sondage!
