# Supplementary material for: Current Use, Capacity, and Perceived Barriers to the Use of Extracorporeal Cardiopulmonary Resuscitation for Out-of-Hospital Cardiac Arrest in Canada
Source: CJC Open. 2020 Nov 13;3(3):327–36. doi: 10.1016/j.cjco.2020.11.005 (PMC7985000; doi:10.1016/j.cjco.2020.11.005)
Supplement: Supplemental Appendix S2 [file mmc2.pdf]

# Canadian ECPR Survey

English version ***La version française suit***

ECPR for Out-of-hospital Cardiac Arrest:EMS Considerations

Thank you for agreeing to participate in this survey! We hope to survey each Canadian emergency medical system region with a cardiovascular surgery capable hospital, to determine practices with regards to ECPR provision for out-of-hospital cardiac arrest. We have sent this survey to the medical lead for prehospital services, however if you believe it is more appropriate to be filled out by another colleague please pass along this survey and email Lindsay.Wilson@blood.ca.

The survey will allow you to exit and return later if you wish.

Please use the following definitions for questions in the survey.

Definitions:

• ECMO: mechanical circulatory support to support heart and/or lung function (includes VA and VV ECMO); does not refer to routine intra-operative management of cardiac surgery

• ECPR: the initiation of VA-ECMO in a patient undergoing active chest compressions during cardiac arrest

• "ECPR for OHCA" fulfills all of the following: (i) the patient had a cardiac arrest in the out-of-hospital setting (ii) ECMO was initiated during active chest compressions (iii) the patient had no periods of sustained ROSC (ie. > 20 min) prior to ECMO initiation (Note: a patient had an OHCA and achieved ROSC which was sustained, then re-arrested during some point after hospital arrival, this is considered ECPR for in-hospital cardiac arrest)

• A formal protocol: A system of care which may include pre-established leadership, protocols, candidacy criteria, designated equipment, and guidelines. Provided the patient meets eligibility criteria, the system is designed to have the capacity to provide the service reliably.

• An ad hoc scenario: The occasional use of ECPR for OHCA within an institution without a formal protocol. Use of ECPR for OHCA may be determined by a case-by-case basis and may not always be available depending on personel and resource availability at the time ECPR is being considered.

Version française ***The English version precedes***

La RCR extracorporelle pour les arrêts cardiaques extrahospitaliers - sondage à l'intention des SMU

Merci d’avoir accepté de participer à ce sondage! Nous espérons sonder le système médical d’urgence de chaque région du Canada où se trouve un hôpital offrant des services de chirurgie cardiovasculaire, afin de déterminer les pratiques relatives à la RCR extracorporelle chez les personnes qui font un arrêt cardiaque en dehors du milieu hospitalier. Nous avons envoyé ce questionnaire au responsable médical des services préhospitaliers. Toutefois, si vous pensez que l’un de vos collègues est mieux placé que vous pour y répondre, veuillez le lui transmettre et envoyer un courriel à Lindsay.Wilson@blood.ca.

Vous pouvez interrompre le sondage en tout temps et y revenir plus tard.

Veuillez vous référer aux définitions suivantes pour répondre aux questions.

Définitions :

• ECMO : technique de circulation mécanique qui offre une assistance cardiaque ou respiratoire [comprend l’ECMO veino-artérielle (ECMO-VA) et l’ECMO veino-veineuse (ECMO-VV)]; ne fait pas référence à la prise en charge peropératoire habituelle de la chirurgie cardiaque.

• RCR extracorporelle : amorce de l’ECMO-VA chez un patient sur lequel on pratique des compressions thoraciques pendant un arrêt cardiaque.

• La définition de la « RCR extracorporelle en cas d’ACEH » englobe tout ce qui suit : (i) le patient a eu un arrêt cardiaque en dehors du milieu hospitalier; (ii) l’ECMO a été amorcée pendant les compressions thoraciques; (iii) le patient n’a eu aucune période soutenue de reprise d’activité circulatoire spontanée (RACS) (c.-à-d. > 20 min) avant le début de l’ECMO. (Remarque : lorsqu’un patient subit un ACEH, qu’il y a reprise de la circulation spontanée mais qu’un autre arrêt cardiaque survient après son arrivée à l’hôpital, le traitement prodigué s’appelle « RCR extracorporelle en cas d’arrêt cardiaque à l’hôpital ».)

• Protocole officiel : système de soins qui peut comprendre une direction préétablie, des protocoles, des critères de sélection, de l’équipement désigné et des lignes directrices. Dans la mesure où le patient répond aux critères d’admissibilité, le système est conçu pour pouvoir offrir le service de façon fiable.

• Situation ponctuelle : utilisation occasionnelle de la RCR extracorporelle lors d’un ACEH dans un établissement qui n’a pas de protocole officiel. Elle peut être déterminée au cas par cas et, selon les ressources et le personnel disponibles au moment où la RCR extracorporelle est envisagée, elle peut ne pas être possible.

## Demographics / Données démographiques

Please enter your name __________________________________

Veuillez inscrire votre nom

What is the name of your emergency medical service (EMS)? __________________________________

Quel est le nom de votre service médical d'urgence (SMU)?

What is your position in the EMS? __________________________________

Quel poste occupez-vous au sein du SMU?

## Regional Characteristics / Caractéristiques régionales

What is the approximate population served by your EMS ? __________________________________

À combien de personnes estime-t-on la population desservie par votre SMU?

Other than your EMS, are there other EMS providers that operate within your region (i.e. that provide the same service you provide)?

À part le vôtre, y a t il d'autres SMU dans votre région (c.-à-d. qui offrent les mêmes services que vous)?

- ◯ Yes / Oui
- ◯ No / Non

How many other EMS agencies are there within your region? __________________________________

Combien y a t il d'autres fournisseurs de SMU dans votre région?

How many hospitals that receive patients by ambulance are located within this region? __________________________________

Combien d'hôpitaux accueillant les patients acheminés par ambulance y a-t-il dans votre région?

How many hospitals have cardiovascular surgery capacity? __________________________________

Combien d'hôpitaux ont un service de chirurgie cardiovasculaire?

How many hospitals in your region perform ECMO? (If unknown, write unknown) ______________________________

Combien d'hôpitaux de votre région pratiquent l'ECMO (si vous ne le savez pas, écrivez >)?

---

## Emergency Medical System (EMS) Characteristics / Caractéristiques du service médical d'urgence (SMU)

How many EMS providers work within your region whose training is limited to BLS resuscitation (not including fire department first responders)? ______________________________

Combien de fournisseurs de SMU dont la formation se□limite aux soins immédiats en réanimation (SIR) - excluant les premiers répondants du service-incendie - travaillent dans votre région?

How many ALS-trained paramedics work in your region? ______________________________

Combien d'ambulanciers ayant une formation en soins avancés en réanimation (SARP) travaillent dans votre région?

Are mechanical CPR devices used in any of the EMS agencies that you are responsible for?

Est-ce que l'un des fournisseurs de SMU dont vous êtes responsable utilise des dispositifs de RCR mécaniques?

- ◯ Yes /Oui
- ◯ No / Non

Which of the following assets are dispatched to an out-of-hospital cardiac arrest in your region? Please select all that apply.

Quelles ressources parmi les suivantes sont-elles dépêchées en cas d'arrêt cardiaque extrahospitalier dans votre région? Cochez toute réponse pertinente.

- ☐ Fire Department first responders / Premiers répondants du service-incendie
- ☐ Bystanders (only include if dispatched by a 9-1-1 operator such as a PulsePoint activation) / Témoins (cochez seulement s'ils sont avisés par un répartiteur du 911 au moyen d'un service d'activation comme PulsePoint)
- ☐ Police / Police
- ☐ EMS responders trained in Basic Life Support / Intervenant d'un SMU formé en SIR
- ☐ EMS responders trained in Advanced Life Support / Intervenant d'un SMU formé en SARC
- ☐ EMS Physician / Médecin d'un SMU

(Select all that apply / Cochez toute réponse pertinente )

Among out-of-hospital cardiac arrests who prove refractory to on-scene therapies, what proportion are then transported to hospital (with ongoing CPR):

Dans quelle proportion les patients ayant un ACEH qui s'avère réfractaire aux traitements administrés sur place sont-ils transportés à l'hôpital (pendant que l'on pratique la RCR)?

- ◯ >90%
- ◯ 50-90%
- ◯ 10-49%
- ◯ < 10%

Please identify the rationale for termination of resuscitation on scene in your region. Please select all that apply.

Veuillez indiquer pourquoi on met parfois fin aux manœuvres de réanimation sur les lieux de l'arrêt cardiorespiratoire dans votre région. Cochez toute réponse pertinente.

- ☐ Do not believe hospital-based treatments have additional benefit to paramedic-led resuscitations / Les soins dans les hôpitaux n'ajoutent rien à ceux prodigués par les ambulanciers
- ☐ Detrimental effect to CPR quality during transport / Effet néfaste sur la qualité de la RCR durant le transport
- ☐ Risk to paramedic safety / Risque pour la sécurité des ambulanciers
- ☐ Risk to public safety / Risque pour la sécurité du public
- ☐ Other / Autre

(Select all that apply / Cochez toute réponse pertinente)

What is the "other" reason you do not transport all to hospital?

Quelle est l'> raison pour laquelle vous ne transportez pas tous les patients à l'hôpital?

__________________________________

Please identify considerations used in your region to determine who is transported with ongoing CPR. Please select all that apply.

Veuillez indiquer comment on détermine qu'un patient sera transporté à l'hôpital pendant que l'on poursuit les manœuvres de RCR. Cochez toute réponse pertinente.

- ☐ Paramedic Discretion / Décision à la discrétion des ambulanciers
- ☐ Physician medical oversight / Supervision médicale d'un médecin
- ☐ The Universal TOR Rule / Règle universelle d'arrêt de la réanimation
- ☐ Those with initial shockable rhythms / Patients ayant un rythme initial justifiant une décharge
- ☐ Those with persistent shockable rhythms / Patients ayant un rythme persistant justifiant une décharge
- ☐ Age / Âge du patient
- ☐ Other criterion / Autre critère

(Select all that apply / Cochez toute réponse pertinente)

What is the other criterion?

Veuillez préciser le autre critère :

__________________________________________

For those transported with ongoing CPR, how many minutes into the resuscitation attempt does this typically occur in your system?

Dans votre système, après combien de minutes de tentative de réanimation décide-t-on généralement de transporter le patient à l'hôpital en continuant les manœuvres de réanimation?

- ◯ 0-15 min
- ◯ 16-30 min
- ◯ > 30 min
- ◯ Other / Autre

Please explain "other"

Si vous avez répondu >, veuillez préciser :

__________________________________________

## Destination Hospital / Hôpital de destination

Is it routine practice for paramedics in your region to provide a pre-alert to the receiving hospital when cardiac arrest patients are en route?

Dans votre région, les ambulanciers ont-ils l'habitude d'avertir l'hôpital de destination lorsqu'un patient en arrêt cardiaque est en route?

◯ Yes / Oui
◯ No / Non

Does the receiving hospital have involvement in overall EMS policies regarding which types of out-of-hospital cardiac arrests who remain pulseless should be transported to hospital?

L'hôpital de destination détermine-t-il en collaboration avec le SMU quels types de patients ayant fait un arrêt cardiaque extrahospitalier (ACEH) et restant sans pouls devraient y être acheminés?

◯ Yes / Oui
◯ No / Non

Are you aware of any receiving hospitals in your region that provide ECPR for OHCA patients on an ad hoc basis or under and established ECPR protocol?

Connaissez-vous un hôpital de votre région qui offre, de façon ponctuelle ou selon un protocole établi, des services de RCR extracorporelle aux patients ayant subi un ACEH?

◯ Yes / Oui
◯ No / Non

Is the ECPR provision for out-of-hospital cardiac arrest within:

Les services de RCR extracorporelle pour les arrêts cardiaques extrahospitaliers sont offerts :

◯ A formal pre-designed protocol (ie. predefined inclusion criteria, designated on-call staff, etc) / dans le cadre d'un protocole préétabli (c.-à-d. critères d'inclusion prédéfinis, personnel en disponibilité désigné, etc.)
◯ Ad hoc provision / de façon ponctuelle
◯ Other / Autre

Please explain "other"

Si vous avez répondu >, veuillez préciser

__________________________________

Do you believe that ECPR may be beneficial for a subset of patients with out-of-hospital cardiac arrest?

Pensez-vous que la RCR extracorporelle pourrait être bénéfique pour un sous-groupe de patients qui auraient fait un arrêt cardiaque extrahospitalier?

◯ Yes / Oui
◯ No / Non

**ECPR Protocol Feasibility**
**Imagine if you were to set up an ECPR protocol in your region where paramedics would assess patients for ECPR eligibility using predefined criteria, pre-alert the hospital for any eligible patients, and then transport the eligible patients with ongoing CPR. Assume that an eligible patient could be identified every 2-8 weeks in your region.**

**Faisabilité d'un protocole de RCR extracorporelle**
**Imaginez que vous deviez établir un protocole de RCR extracorporelle dans votre région. En vertu de ce protocole, les ambulanciers évalueraient l'admissibilité des patients à la réanimation cardiorespiratoire extracorporelle au moyen de critères prédéfinis, puis ils informeraient l'hôpital de l'arrivée de tous patients admissibles et y achemineraient ces patients en poursuivant les manœuvres de réanimation. Supposez qu'un patient admissible pourrait être identifié toutes les deux à huit semaines dans votre région.**

| | |
|---|---|
| Do you think such a protocol would be feasible?<br><br>Pensez-vous qu'un tel protocole serait réalisable? | ◯ Yes / Oui<br>◯ No / Non |

**What would be the barriers to implementing such a protocol?**

**Indiquez dans quelle mesure les éléments suivants seraient des obstacles à la mise en œuvre d'un tel protocole.**

| | Not a Barrier / Aucun obstacle | Small Barrier / Obstacle mineur | Moderate Barrier / Obstacle modéré | Large Barrier / Obstacle important | Very Large Barrier / Obstacle majeur |
|---|---|---|---|---|---|
| EMS leadership does not believe the therapy works / La direction du SMU ne croit pas que le traitement fonctionne | ○ | ○ | ○ | ○ | ○ |
| There is no robust evidence proving the therapy leads to survivors / Il n'existe pas de données fiables qui prouvent que le traitement sauve des vies | ○ | ○ | ○ | ○ | ○ |
| Paramedic training / La formation des ambulanciers | ○ | ○ | ○ | ○ | ○ |
| Paramedic expertise in patient identification / L'expertise des ambulanciers à reconnaître ces patients | ○ | ○ | ○ | ○ | ○ |
| Low volume of patients would be a barrier to EMT/paramedic competency / Le faible volume de patients serait un obstacle au développement des compétences des techniciens médicaux d'urgence (EMT) ou des ambulanciers | ○ | ○ | ○ | ○ | ○ |
| Medical leadership would deem the detrimental effect of transport in excess of the potential gain of ECPR / Selon les autorités médicales, l'effet néfaste du transport serait supérieur aux bienfaits possibles de la RCR extracorporelle | ○ | ○ | ○ | ○ | ○ |
| Lack of co-ordination between prehospital and hospital based services / Le manque de coordination entre les services préhospitaliers et les services hospitaliers | ○ | ○ | ○ | ○ | ○ |

| | | | | | |
|---|---|---|---|---|---|
| The hospital costs and clinical resources required do not justify the potential survival benefits / Les coûts hospitaliers et les ressources cliniques nécessaires ne justifient pas les bénéfices potentiels pour la survie | ○ | ○ | ○ | ○ | ○ |
| Other / Autre | ○ | ○ | ○ | ○ | ○ |

Discuss what you meant by "other"

Si vous avez répondu >, veuillez préciser de quoi il s'agit ci-dessous et indiquer ci-dessus dans quelle mesure cet élément représente un obstacle : __________________________________
