# Supplementary material for: Current Use, Capacity, and Perceived Barriers to the Use of Extracorporeal Cardiopulmonary Resuscitation for Out-of-Hospital Cardiac Arrest in Canada
Source: CJC Open. 2020 Nov 13;3(3):327–36. doi: 10.1016/j.cjco.2020.11.005 (PMC7985000; doi:10.1016/j.cjco.2020.11.005)
Supplement: Supplemental Appendix S3 [file mmc3.pdf]

Supplemental Appendix S3: Survey Respondents

Hospitals

1. Alberta Children's Hospital, Calgary, Alberta
2. BC Children's Hospital, Vancouver, British Columbia
3. Centre Hospitalier de L`Universite de Montreal (CHUM), Montreal, Quebec
4. Centre hospitalier universitaire Sainte-Justine, Montreal, Quebec
5. Centre intégré universitaire de santé et de services sociaux (CIUSSS) - Centre Hospitalier Univeritaire de Sherbrooke (CHUS), Sherbrooke, Quebec
6. Centre intégré universitaire de santé et de services sociaux (CIUSSS) du Saguenay–Lac-Saint-Jean Installation Chicoutimi, Chicoutimi, Quebec
7. Foothills Medical Centre, Calgary, Alberta
8. Glen Hospital – McGill University Health Centre, Montreal, Quebec
9. Halifax Infirmary – Queen Elizabeth II Health Sciences Centre, Halifax, Nova Scotia
10. Hamilton General Hospital, Hamilton, Ontario
11. Health Sciences Centre – General Hospital – Eastern Health, St. John’s, Newfoundland and Labrador
12. Health Sciences North, Sudbury, Ontario
13. Hôpital du Sacré-Coeur de Montréal, Montreal, Quebec
14. Izaak Walton Killam Hospital for Children, Halifax, Nova Scotia
15. Jewish General Hospital, Montreal, Quebec
16. Kelowna General Hospital, Kelowna, British Columbia
17. Kingston Health Sciences Centre, Kingston, Ontario
18. London Health Sciences Centre, London, Ontario
19. Montreal Children's Hospital, Montreal, Quebec
20. Regina General Hospital, Regina, Saskatchewan
21. Royal Columbian Hospital, New Westminster, British Columbia
22. Royal Jubilee Hospital, Victoria, British Columbia
23. Royal University Hospital, Saskatoon, Saskatchewan
24. Saint John Regional Hospital – Horizon Health Network, Saint John, New Brunswick
25. St. Michael's Hospital, Toronto, Ontario
26. Southlake Regional Health Centre, Newmarket, Ontario
27. St. Boniface Hospital, Winnipeg, Manitoba
28. St. Mary's General Hospital, Kitchener, Ontario
29. Stollery Children's Hospital, Edmonton, Alberta
30. St. Paul's Hospital, Vancouver, British Columbia
31. Sunnybrook Health Sciences Centre, Toronto, Ontario
32. The Hospital for Sick Children, Toronto, Ontario
33. The Montreal Heart Institute, Montreal, Quebec
34. Trillium Health Partners, Mississauga, Ontario
35. University Health Network, Toronto, Ontario

36. University Institute of Cardiology and Pneumology of Quebec - Laval University, Quebec City, Quebec
37. University of Alberta, Edmonton, Alberta
38. University of Ottawa Heart Institute, Ottawa, Ontario & Children's Hospital of Eastern Ontario, Ottawa, Ontario (these hospitals submitted data as a single institution)
39. Vancouver General Hospital, Vancouver, British Columbia

Emergency Medical Services

1. Alberta Health System Emergency Medical Services, Alberta
2. Ambulance New Brunswick, New Brunswick
3. British Columbia Emergency Health Services, British Columbia
4. Corporation of Emergency Medical Technicians Quebec, Quebec
5. Emergency Health Services Nova Scotia, Nova Scotia
6. Frontenac Paramedics, Ontario
7. Hamilton Paramedic Service, Ontario
8. Leeds Grenville Paramedic Service, Ontario
9. Lennox and Addington, Ontario
10. Medavie Health Services West, Saskatchewan
11. Metro Paramedic Service, Newfoundland and Labrador
12. Middlesex-London Paramedic Service, Ontario
13. Ottawa Paramedic Service, Ontario
14. Peel Regional Paramedic Services, Ontario
15. Regina Queppelle Health Region, Saskatchewan
16. Region of Waterloo Paramedic Services, Ontario
17. Sudbury Emergency Medical Services, Ontario
18. Toronto Paramedic Services, Ontario
19. Urgences-santé Quebec, Quebec
20. Winnipeg Fire Paramedic Service, Manitoba
21. York Region Paramedic Services, Ontario
