# Supplementary material for: Current Use, Capacity, and Perceived Barriers to the Use of Extracorporeal Cardiopulmonary Resuscitation for Out-of-Hospital Cardiac Arrest in Canada
Source: CJC Open. 2020 Nov 13;3(3):327–36. doi: 10.1016/j.cjco.2020.11.005 (PMC7985000; doi:10.1016/j.cjco.2020.11.005)
Supplement: Supplementary Materials [file mmc4.pdf]

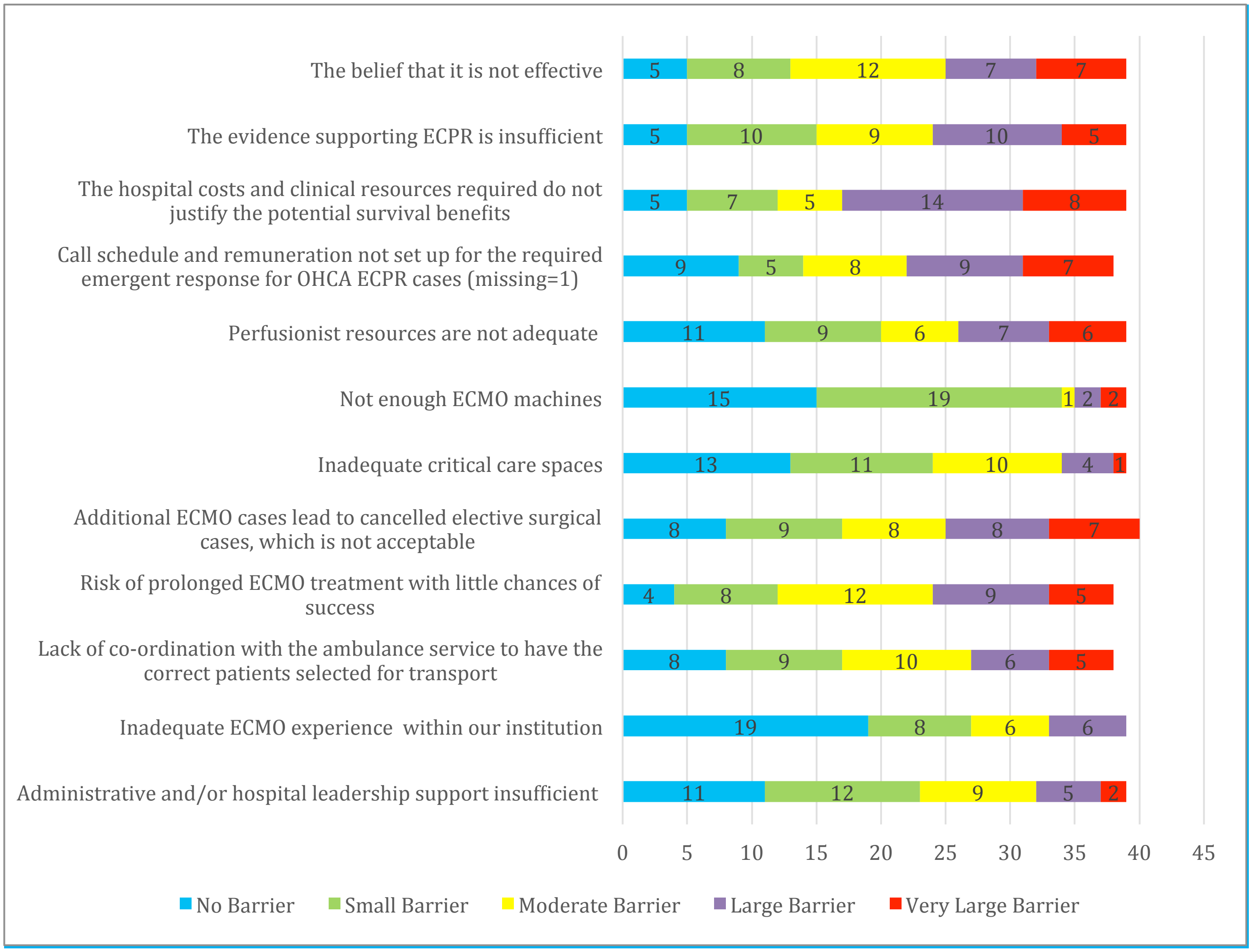

Suppl. Figure S1: Perceived barriers to the provision of ECPR for out-of-hospital cardiac arrest from 39 hospital-based respondents

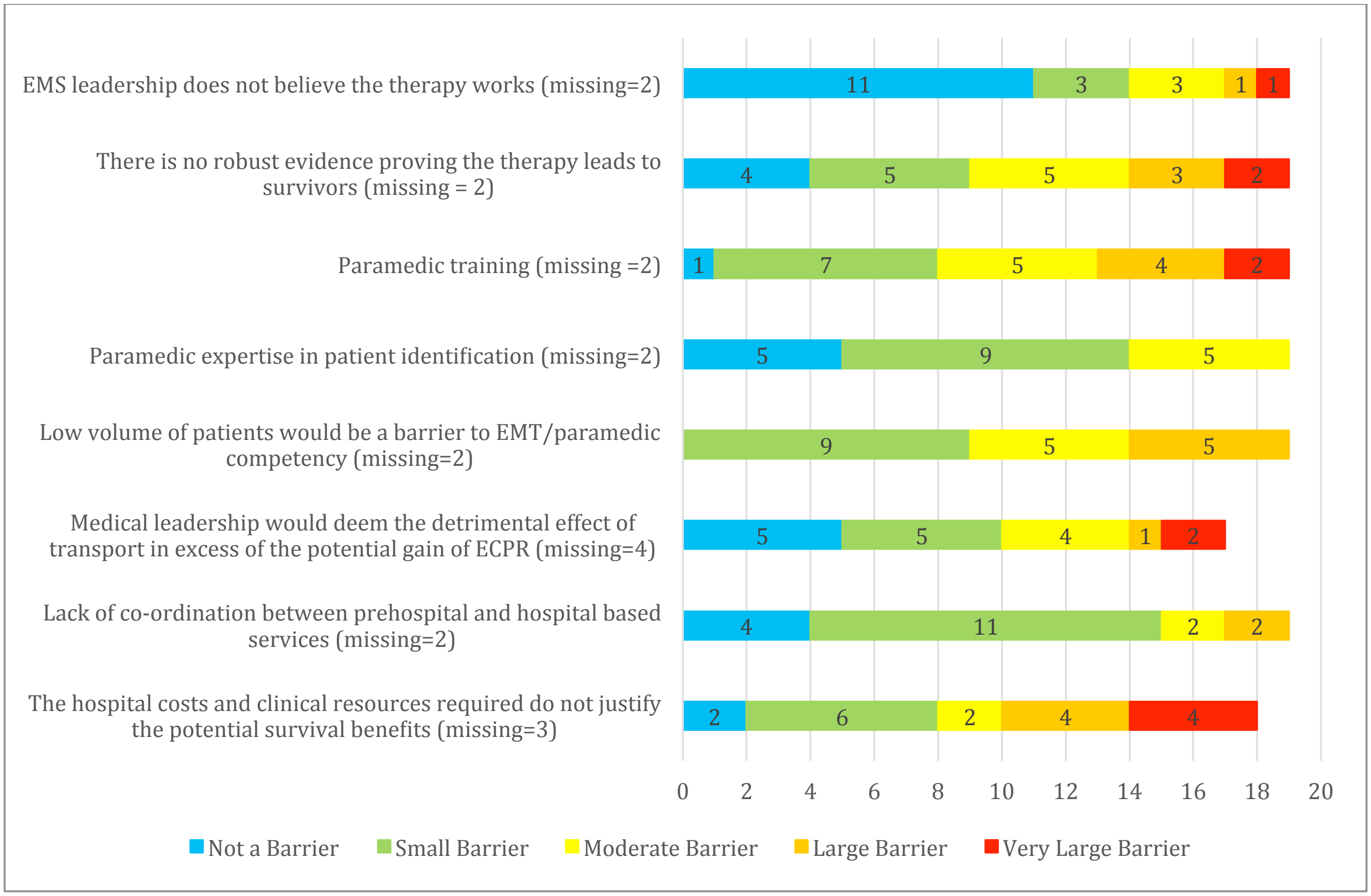

Suppl. Figure S2: Barriers to Implementing an ECPR Protocol from 21 EMS-based respondents
